# Access to public drinking water fountains in Berkeley, California: a geospatial analysis

**DOI:** 10.1186/s12889-018-5087-4

**Published:** 2018-01-24

**Authors:** Dylan C. Avery, Charlotte D. Smith

**Affiliations:** School of Public Health, University of California Berkeley, 50 University Hall #7360, Berkeley, CA 94702 USA

**Keywords:** GIS, Spatial analysis, Demographics, Water, Fountains, Beverage tax

## Abstract

**Background:**

In January 2015, Berkeley, California became the first city in the Unites States to impose a tax on sugar-sweetened beverages. The tax is intended to discourage purchase of sugary beverages and promote consumption of healthier alternatives such as tap water. The goal of the study was to assess the condition of public drinking water fountains and determine if there is a difference in access to clean, functioning fountains based on race or socio-economic status.

**Methods:**

A mobile-GIS App was created to locate and collect data on existing drinking water fountains in Berkeley, CA. Demographic variables related to race and socio-economic status (SES) were acquired from the US Census – American Community Survey database. Disparities in access to, or condition of drinking water fountains relative to demographics was explored using spatial analyses. Spatial statistical-analysis was performed to estimate demographic characteristics of communities near the water fountains and logistic regression was used to examine the relationship between household median income or race and condition of fountain.

**Results:**

Although most fountains were classified as functioning, some were dirty, clogged, or both dirty and clogged. No spatial relationships between demographic characteristics and fountain conditions were observed.

**Discussion:**

All geo-located data and a series of maps were provided to the City of Berkeley and the public.

**Conclusions:**

The geo-database created as an outcome of this study is useful for prioritizing maintenance of existing fountains and planning the locations of future fountains. The methodologies used for this study could be applied to a wide variety of asset inventory and assessment projects such as clinics or pharmaceutical dispensaries, both in developed and developing countries.

## Background

Recent studies suggest a link between consumption of sugary beverages and adverse health outcomes such as diabetes, obesity, and tooth decay [1, 2]. In many urban centers, drinking water fountains are available to the public as free and healthy alternatives to sugar-sweetened beverages. In Berkeley, California, drinking water fountains can be found in schools, parks, and other public and semi-public spaces. The public perception of drinking water fountains in school environments has been well documented [3, 4]. Patel et al. observed that although schools generally have abundant accessible fountains, varying public perceptions of fountains influences fountain utilization [4]. Because there are differences in perception of fountains in the school environment, where accessibility is constant, the perception of drinking fountains may be linked to a disparity in access and condition of fountains outside of the school environment as well.

Additionally minority and low socio-economic populations, who report lower accessibility to fountains [5], are less likely to drink from free drinking fountains, and more likely to choose more expensive bottled water [3]. This raises the questions that this study aimed to answer: Is there a disparity in access to, or condition of drinking water fountains relative to race and socio-economic status (SES) in Berkeley California? While this case describes the situation in a specific city, the question is generalizable to all cities which have public water fountains. Additionally, the methodology to answer this question is applicable to other assets (for example, health clinics or pharmaceutical dispensaries).

Several studies have explored the relationship between population demographic characteristics and access to community resources [6–8]. These studies differed in their findings, which could be due to their differing methodologies. Each study noted that there was a positive association between access to community resources and positive health outcomes. However, there is disagreement in what combination of community resources should be used as a metric for area deprivation, or observable disadvantage [9]. For example, using the metric: “number of accessible parks” as the measurement of access, Cutts et al. claimed that communities with higher access to parks appeared to have worse health outcomes [8]. However, Boone et al. reported a direct relationship between access and positive health outcomes when using the number of parks or acreage as a metric for accessibility, while controlling for race. Although there can be adequate access to community resources by absolute number, there still may be disparities when measuring accessibility as acreage [7]. The conflicting conclusions about the relationship between quality/condition and health outcomes based on the criteria used to describe access and condition shows that it is important to carefully consider what criteria for access and quality most accurately describes the true relationship without bias. As access to community resources has a connection with health, identifying locations with disparities in access to resources (such as drinking water) and observing their relationship with SES and race is valuable.

While analyzing the distribution of race and SES is useful, it is also important to consider location as a factor. Analyzing spatial patterns can provide further insight into the complex relationship between access to community resources and health [10]. Proximity, density, or a combination of the two, are common measurements used to describe the environment spatially [11]. Several studies [6, 12, 13] used geospatial information systems (GIS) to successfully identify disparities in access to community resources at a regional level, but acknowledge the difficulty of observing a relationship at a local scale.

This study aimed to explain the relationship between SES and race with access to drinking water fountains of different conditions. Two hypotheses guided this study: 1) there is a relationship between the spatial distribution of drinking water fountains with SES or race and 2) that there is a relationship between the condition of drinking water fountains with SES or race. Correlation coefficients and GIS technology were used to observe the relationship between drinking water fountains with SES or race at a local level in Berkeley, California. This study quantitatively examined access to fountains spatially, and compared multiple indicators of drinking water fountain condition to surrounding demographic characteristics.

## Methods

Data fields were designed to achieve a balance between informative characterization of the fountain and practical length. A total of 29 volunteers were recruited to collect data on fountains in the city of Berkeley. A mobile-phone data-collection software platform (mobile phone App) was used, instead of traditional paper forms, for more efficient data collection and elimination of transcription errors. When possible, data fields were designed to be dichotomous to avoid bias among volunteers. Several variables were collected automatically by the software (AmigoCloud, San Francisco, CA), such as a unique observation ID number, date, time, and geographic coordinates (latitude and longitude). The volunteers also manually recorded location for validation. Additional variables included fountain type, condition (functioning status and cleanliness of the fountain), ability to fill a water bottle, wheelchair accessible, comments and photos. The volunteers looked for ramps and observed the accessibility of the surrounding terrain in order to determine accessibility.

Any fountains that were clogged (not draining water properly), leaking, littered, splashing or had a strange smell were recorded. As a measure of desirability to drink from the fountain, the stream height of each fountain was recorded as greater than 4 in. above the fountain spout, less than 4 in., less than spout, and no arc at all.

**Fig. 1:**
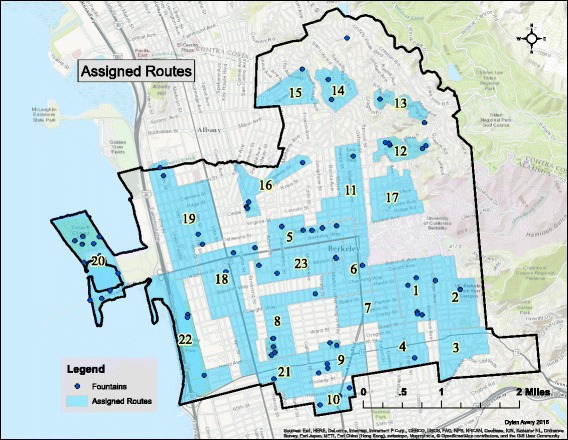
Assigned Routes and Fountain Locations

Geographic information system tools and methods were used to create a survey area to achieve a high proportion of fountains, given a limited number of volunteers. ArcMap 10.2.2 (ESRI, Redlands, CA) was used to create a geodatabase with survey areas and existing fountain locations (based on information from the City of Berkeley’s Parks and Recreation Department). Shapefiles of city streets, parks, zoning, and city boundaries were provided by the City of Berkeley Information and Technology Department [14]. The hand drawn locations of existing fountains were geocoded into a shapefile in the prepared geodatabase using ArcMap. Polygons were drawn around all geocoded fountain points and around areas with high pedestrian traffic. Residential zones without any geocoded fountain points were excluded from the survey-area polygons, as there would not likely be any fountains in these areas. Areas surrounding parks and near bicycle paths were included in the survey-area polygons as these areas were hypothesized to have drinking fountains near them. A total of 24 survey-area polygons were designed that covered all areas that possibly contain drinking water fountains within the City and are in Fig. 1.

A non-random surveyor assignment method was chosen because the volunteer surveyors were students, which in general had transportation restrictions (i.e. most without access to cars). With a random assignment, it would be likely that several surveyors would be assigned to a location that was inaccessible to them. The students were shown all survey-areas on a map of the City of Berkeley (Fig. 1) and chose three preferred survey-areas. Surveyors were then assigned to one of their preferred area. No more than two people were assigned to each survey-area. A non-random assignment was not likely to result in bias in this study.

All statistical analysis was performed in RStudio using R programming language (RStudio, Boston, MA). The statistical analysis was written in an R markdown document format. GitHub™ was used as the version control system for the dataset and coding for statistical analysis. ArcMap 10.2.2 was also used in the exploratory data analysis and to perform a spatial join of the fountain locations with demographic data. Demographic data was collected from the U.S. Census TIGER database as shapefiles with demographic attribute data from the 2009–2013 American Community Survey (ACS). City boundary and streets, provided by the City of Berkeley, were used as a basemap for the choropleth maps.

**Fig. 2:**
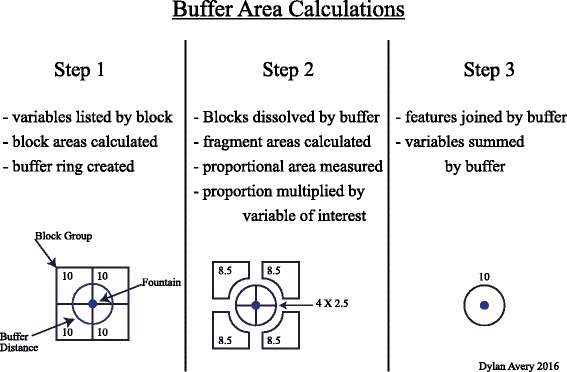
Buffer Area Calculation

In order to achieve an accurate estimation of the demographic characteristics surrounding each fountain, several buffer rings at multiple distances surrounding each fountain were used. Each block group’s total area (meters squared) was calculated in ArcMap using NAD 1983 2011 UTM Zone 10 N as the projected coordinate system. Total areas were calculated using the 2015 census block groups that were clipped to the City of Berkeley land boundaries. As a result of clipping, some buffers protrude over the city boundaries into areas with no demographic data. The demographic characteristics of the regions within each buffer zone were estimated using the portion of the areas with demographic data (Fig. 2). No significant bias was likely introduced since the buffer distances are smaller than the ACS block groups and the study was conducted at a local scale such that variances in demographic features are minimal. The proportion of each block group that each buffer ring covered was calculated. Next the demographic characteristics of interest for each block group was multiplied by its corresponding proportional area contained by each buffer area. Then the proportional demographic values for each fountain buffer distance were summed. The last step was to normalize the summed demographics of all buffers to the area of the smallest buffer zones (19,300 m^2^). Some buffer areas reached beyond the city boundaries and were not able to join with demographic data and were omitted from data analysis. Buffers within the Aquatic Park and Berkeley Marina were also excluded from data analysis since only a vey small population resides there, according to census data.

The method described above estimated the demographic characteristics within a given diameter centered at a fountain, rather than by the closest block group. Three buffer ring distances were used in this analysis. The mean area of block groups for the City of Berkeley was calculated (μ = 19,300 m^2^, *δ*=14,600 m^2^) and used to determine the smallest buffer distance. The average block group area was equivalent to an area of a buffer with a radius of roughly 80 m. The radius of each ring increased by the equivalent of one standard deviation of the distribution of block group areas in Berkeley (radius about 70 m) for a total of three different buffer distances. Information on the sizes of all buffer distances can be seen in Table 1. Because the distribution of survey-areas was right skewed and included many block groups clipped by the city boundaries and have very small areas (1,000,000-fold variance of block group areas), only the middle 90% of the distribution of block group areas were used in calculating the mean and standard deviation of block group areas (100-fold variance of block group areas). Since the unit of area is m^2^, fold-ranges more intuitively described the variance of areas than other measurements of range.

**Table 1:** Buffer ring sizes

Buffer Radius (m)	Buffer Area (m^2^)	Relative Size
80	20,100	1
150	70,650	0.284
220	152,000	0.132

Descriptive statistics and spatial visualizations were performed on each explanatory variable. Collected data on metrics of fountain condition (e.g. clean/dirty) were used as outcome variables. Census demographic data (i.e. median income or race) were used as explanatory variables. Thematic maps of the block group demographics with point features of each fountain overlaid were also produced in ArcMap. These maps provide a spatial visualization of the descriptive statistics.

In order to perform regression analysis, the assumption that residual values are independent and randomly distributed must be reasonably observed in the dataset [15]. Where spatial autocorrelation is present and residuals are not independent, this assumption is violated [16]. Spatial autocorrelation was assessed using Moran’s I test, and local cluster analysis was performed using the Anselin Local Moran’s I statistic.

Generalized linear models were used to explore the relationship between each outcome variable (condition) and the explanatory variables (demographics). Dichotomous outcome variables included *clean, functioning, clogged, littered, leaking, and splashing*. Polytomous outcome variables include water taste and stream height. Similar to other studies on access to public resources, household median income was used to describe socio-economic status [7, 8]. American Community Survey 2014 demographic data on median household income and proportions of racial groups were used as explanatory variables in the models. The relationship between race and median income was also described. A data transformation of the explanatory variables was necessary in order to achieve a normal distribution for regression analysis. In this case, the square root of median household incomes was used. Collinearity between the estimates of median income and each proportional race at each buffer distance was measured (Fig. 7).

**Fig. 3:**
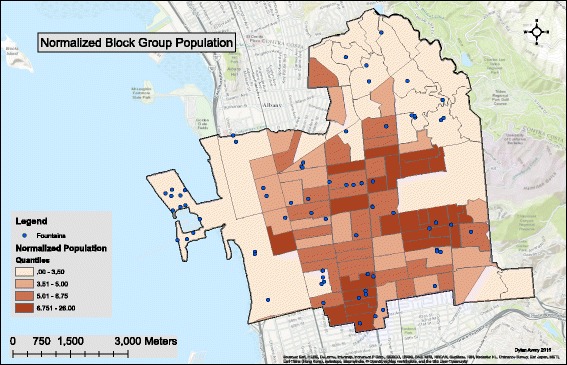
Population Normalized by Block Group

As seen in Fig. 3, the western region of the city of Berkeley contains a relatively small population density. This is due to the fact that this region is mostly zoned as manufacturing, similarly purposed zones, or parks (Fig. 1). Because of this, statistical analysis was performed without fountains existing in low density block groups (defined as < 30 people). Sixteen fountains from these areas were omitted from the data analysis. Census data on race by block group was normalized before any inferential statistics were performed due to variance in the total population by block group, and variance in the size of each block group.

### Map design

In this study, an emphasis was placed in the communication of data and results through map design. A series of reference, thematic and web maps were produced for the benefit of the public. Reference maps to be used by the city of Berkeley, were produced in ArcMap. A series of thematic maps were produced for the public using ArcMap, Mapbox (Mapbox, San Francisco, CA) and Adobe Illustrator (Adobe, San Jose, CA). Vector tiles were produced in Mapbox Studio using CartoCSS (CartoDB, New York, NY) language, and used as base maps for the thematic maps. Reference maps were produced in ArcMap and were used as a framework for the thematic maps. The thematic maps were designed primarily in Adobe Illustrator. The interactive web map was produced using ArcGIS online software.

## Results

Descriptive statistics of the fountain data was performed to guide the spatial data analysis. The variables collected that were explored in the analysis included; Fountain Type (factor), clean (y/n), clogged (y/n), functional (y/n), handicap accessible (y/n), leaking (y/n), littered (y/n), odor (y/n), rusted (y/n), splashing (y/n), taste (factor), water bottle accessible (y/n). Variables such as fountain type and handicap accessibility are not direct measures of fountain condition, however they provide more insight into the other variables that describe condition.

### Fountain condition

**Fig. 4:**
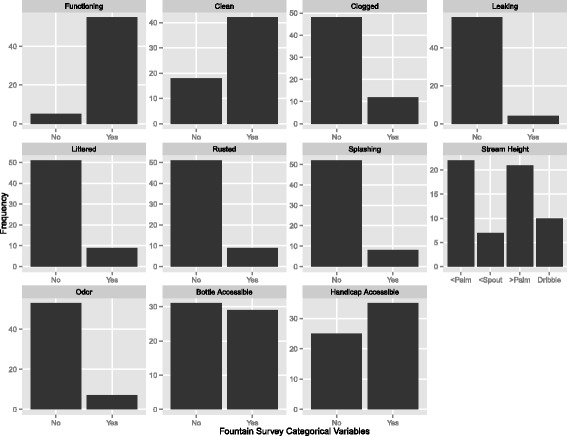
Surveyed Fountain Characteristics

Sixty fountains were included in the statistical analysis. Raw counts of each variable collected in the fountain survey are provided in Table 2. An overwhelming majority of fountains (92%) were classified as functioning. Also, several other variables had a low rate of poor fountain condition (leaking, littered, rusted, splashing, and odor). Highly contrasted outcomes for categorical variables affects the strength of statistical analysis with a small sample size. Several variables were not used as they had a low frequency (Fig. 4).

**Table 2:** Summary of fountain survey categorical variables

	Functioning	Clean	Clogged	Leaking	Littered	Rusted	Splashing	Odor	Refill^a^	Handicap^b^
No	5	18	48	56	51	51	52	53	31	25
Yes	55	42	12	4	9	9	8	7	29	35

^a^Refill = Ability to fill a standard water bottle

^b^Handicap Accessibility

However, some variables were not severely unbalanced. There was nearly an equal distribution of different fountain stream heights. Thirty-five percent of fountains had a stream height of above 4 in. and 36% had a reported height of above the spout height but lower than 4 in.. The remaining 28% were reported to have a stream height of less than the fountain spout. Of those with a stream height of less than the fountains spout, 58% had no stream arc (dribbling in contact with fountain spout at all times).

**Fig. 5:**
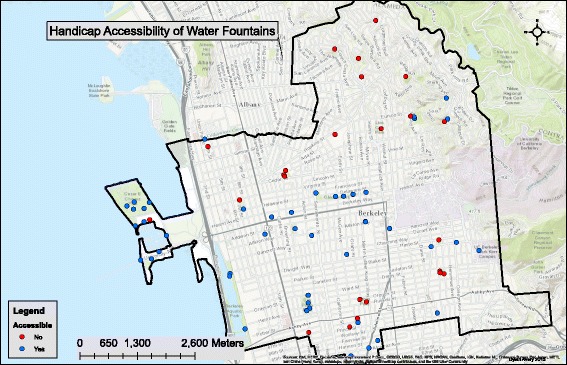
Fountains and Handicap Accessibility

There was no evidence of significant spatial patterns or clustering in analyzing the spatial distribution of each indicator of fountain condition except for handicap accessibility. Of the surveyed fountains, 58% were recorded as being handicap accessible. As evident in Fig. 5, there was some clustering of handicap accessible fountains in the western region of Berkeley and some clustering of non-handicap accessible fountains in the northern region of Berkeley. The clustering of no-handicap accessibility is present in a region with steep slopes. The inverse is seen in the western region where there is a relatively small elevation change.

Sixty-two percent of recorded fountains used in the analysis were not compatible with refilling a standard sized water bottle (defined as 9 in.). This could have been due to several factors such as a low spout height, inconveniently shaped basin, or a combination of the two conditions. As indicated in Table 3, of all fountains observed to have a stream height of more than the surveyor’s 4 in., most (86%) were reported to be accessible for typical water bottles. Less than half of the fountains (46%) were accessible for water bottles if the stream height was less than the surveyors 4 in. but higher than the spout itself. Only one of the 17 fountains with a stream height of less than the spout was reported to be accessible for water bottle refill, due to a unique fountain design. Fountain stream height and water bottle accessibility had a Pearson’s Chi-Squared coefficient of 24.3 (*p* = 0.002), indicating these variables were positively correlated.

**Table 3:** Fountain stream height and water bottle accessibility

Height	Accessible	Not Accessible	Total
> 4 in.	18	3	21
< 4 in.	10	12	22
<Spout	0	7	7
Dribble	1	9	10
Total	29	31	60

**Fig. 6:**
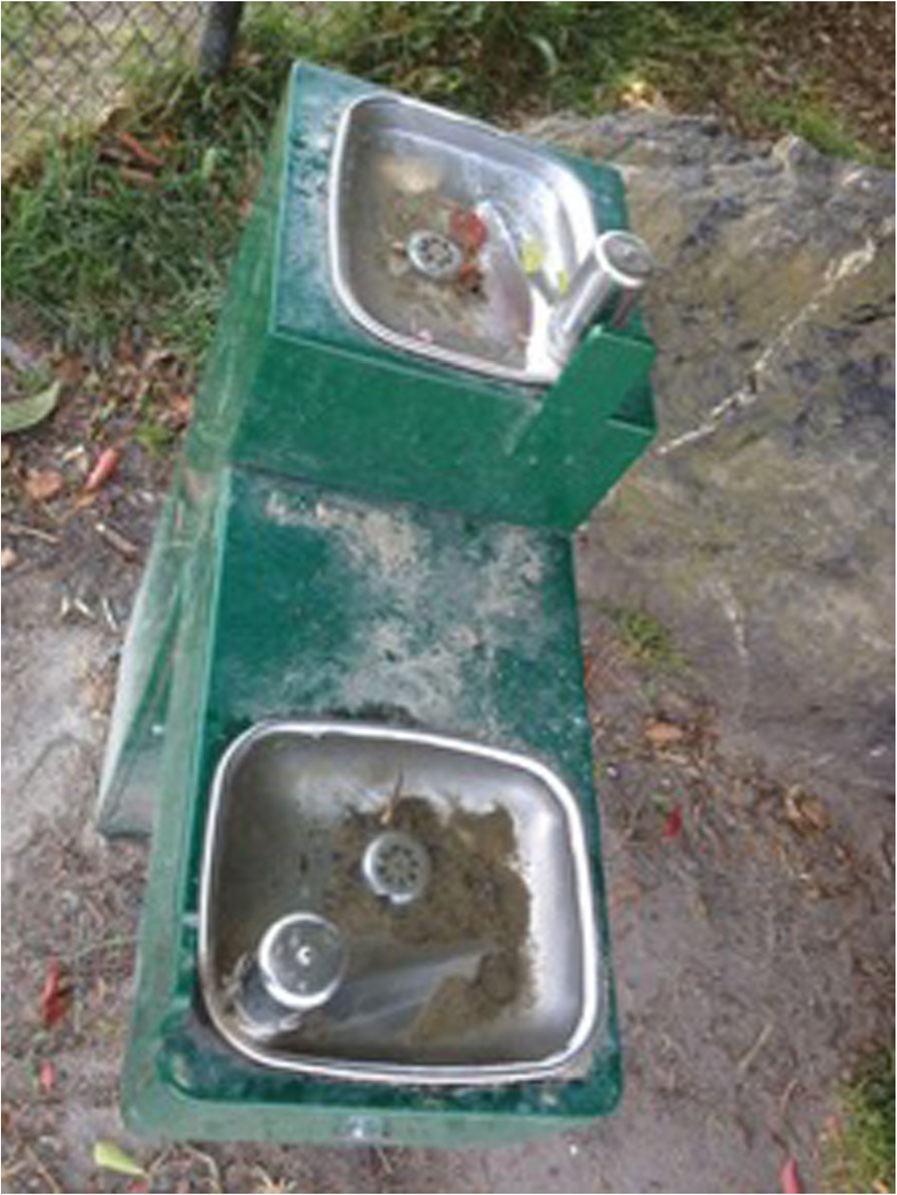
Picture of Surveyed Fountain

As shown in Tables 4, 70% of the fountains were reported to be clean. Fig. 6 shows a fountain that is not clean. Twenty percent (19) of the fountains were reported to be clogged. Of those that were recorded as clean, only 4 (10%) were also reported to be clogged. The relative risk of a fountain being clogged if it were not clean was 4.6 (95% CI: 1.6–13.6). The relative risk was 3.2 (95% CI: 1.6–6.3) for a fountain being not clean if it were clogged. The Pearson’s Chi-Squared coefficient for fountain cleanliness and clogged status was 9.6 (*p* = 0.04). At the *p* < 0.05 threshold, independence between fountain cleanliness and clogged status was rejected.

**Table 4:** Summary of clean vs clogged fountains

	Not Clean	Clean	Total
Clogged	8	4	12
Not Clogged	10	38	48
Total	18	42	60

### Socio-economic status

Median household incomes were calculated for each buffer distance. The distribution of median incomes appeared to be fairly similar between the different buffer distances. There was a slight increase in central tendency of the median income values as the buffer distance increases. It was also evident that the distribution of median incomes was skewed to the right indicating a disproportionate number of high income households.

### Race

American Community Survey (ACS) data provided estimates of the absolute number of self-reported race by block group. The 2015 ACS data indicated that of Berkeley’s 117,384 residents, 23.5% were Asian, 9.30% were Black, 67.7% were White. Approximately 7% of the population was comprised of other races including American Indian, Alaska Native, Native Hawaiian. The percent of each reported race (Asian, Black, and White) was estimated for each buffer area around each fountain (radius: 80 Meters, 150 Meters and 220 Meters). Within each racial group there is an increasing central tendency of the percent race as buffer distance increases. Although the distributions have similar structure across races, overall the percent of reported as white was much higher than other races for all buffer distances.

**Fig. 7:**
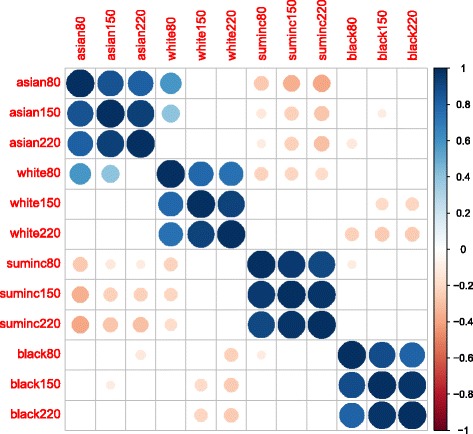
Income and Race Correlogram

Correlation between buffer distances for each explanatory variable was significant (*p* < 0.05). There was no evidence of a significant relationship between different racial groups within the two lower buffer distances (Fig. 7). There was a significant negative relationship between the estimated proportion of reported as white and reported as Asian at the 80-m buffer distance (Fig. 7). There was also a significant negative relationship between the estimated proportion of reported as white and reported as black at both the 150-m and the 220-m buffer distance (Fig. 7). This was expected as the measure was normalized on the same total population. At each buffer distance, there was a significant negative relationship between household median income and proportion of reported as Asian. As this proportional relationship can be demonstrated in the calculated buffers, the use of one subset (rather than all) which best fits model assumptions is appropriate.

### Regression

Logistic regression was performed to explore relationships between fountain conditions and demographic characteristics. Cleanliness, clogged status, bottle accessibility, and handicap accessibility were used as outcome variables. Median household income and proportion of reported as white was used as explanatory variables. The reported as white was used instead of the other ethnicities because there was minimal collinearity between reported as white and the other explanatory variables. There were no strong associations between median household income or reported race and fountain conditions. There was a significant association between median income and handicap accessibility at the 80-m buffer distance, however, the relationship is not linear (R coefficient of − 0.00, *p* = 0.04). There was a negative relationship between handicap accessibility and income at the most local level used in this study (Tables 5, 6 & 7), however this may be explained by the spatial clustering in certain regions of Berkeley due to topological slope, since clustering of non-handicap accessible fountains occurred in a higher income region. In Berkeley, the high-priced homes are in the hills.

**Table 5:** Bivariate logistic regression summary | 80 meter features

	Estimate	Standard Error	Z Value	P(>|Z|)
Clean and Income	−0.00	0.00	− 0.75	0.45
Clean and White	−0.95	1.52	−0.63	0.53
Clogged and Income	0.00	0.00	0.85	0.40
Clogged and White	0.64	1.70	0.38	0.70
Bottle and Income	−0.00	0.00	−0.89	0.37
Bottle and White	−0.31	1.47	−0.21	0.83
Handicap and Income	−0.00	0.00	−2.09	0.04
Handicap and White	1.42	1.65	0.86	0.39

**Table 6:** Bivariate logistic regression summary | 150 meter features

	Estimate	Standard Error	Z Value	P(>|Z|)
Clean and Income	−0.00	0.00	−1.05	0.30
Clean and White	−1.56	3.61	−0.43	0.67
Clogged and Income	0.00	0.00	0.95	0.34
Clogged and White	2.82	3.86	0.73	0.46
Bottle and Income	−0.00	0.00	−0.82	0.41
Bottle and White	1.36	3.48	−0.39	0.70
Handicap and Income	−0.00	0.00	−1.79	0.07
Handicap and White	−4.23	3.72	−1.14	0.26

**Table 7:** Bivariate logistic regression summary | 220 meter features

	Estimate	Standard Error	Z Value	P(>|Z|)
Clean and Income	−0.00	0.00	−1.02	0.31
Clean and White	−5.33	9.35	−0.57	0.57
Clogged and Income	0.00	0.00	1.08	0.28
Clogged and White	11.51	10.22	1.13	0.26
Bottle and Income	−0.00	0.00	−1.23	0.22
Bottle and White	−1.96	8.84	−0.22	0.82
Handicap and Income	−0.00	0.00	−1.66	0.10
Handicap and White	13.47	9.28	−1.45	0.15

## Discussion

Several assumptions were used to redefine the spatial boundaries of the demographic data. Before intersecting boundaries, the demographics within each block group were assumed to be homogeneous. Additional assumptions were made in calculating median household income around fountains since raw data on individual household incomes is not available. A government data base that aggregates individual household income in a neighborhood was used for this study (in this case, the “block group”). The local scale of the analysis minimizes any potential bias. Since census data was clipped to the City of Berkeley’s boundaries, some buffer zones were excluded. There is no quantitative data available to measure the amount of sampling error explained by different sources, 95% of surveyed fountains were within 76 m of fountain locations provided by the City of Berkeley. Because this variance is lower than our smallest buffer distance, there is not a high concern of misclassifying fountains with block group attributes in the statistical analysis.

In the initial analysis, most drinking water fountains in the City of Berkeley were classified as functioning. Functioning status is a good indicator of whether or not a fountain can be used. Further, it was also essential to examine other indirect indicators of condition to provide insight into the chances a fountain will be used. For example, the data showed that although at the time of survey over 90% of drinking water fountains were functioning, roughly 30% were classified as not clean and 20% were classified as clogged. The clean and clogged status of the fountains reported in this study has a significant level of dependence. The spatial distribution of drinking water fountains along with their condition was also explored. In areas with high pedestrian traffic in Berkeley, sugary beverage products provided by vendors are more accessible than fountains. There were no observable spatial patterns based on fountain cleanliness or clogged status. This suggests that although many fountains are functioning, they might not actually be used frequently since many were dirty and/or clogged. This study did not include observations of drinking water fountain usage, or water quality as those measures were beyond the scope and budget of the project.

In the City of Berkeley (excluding the University campus and private properties), only one drinking water fountain was reported to be developed specifically for refilling reusable water bottles (sometimes known as hydration stations). The feature that distinguishes hydration stations from typical drinking water fountains is how the water stream is designed to flow. Fountains designed for direct consumption have a distinct arc, first shooting up out of the spout. Fountains designed for refilling water bottles typically have a stream that flows straight down towards a drain and is intended for filling a bottle rather than drinking by placing one’s mouth in the stream. Only 38% of the fountains surveyed were reported to be water bottle refillable. There was no observable spatial pattern in water bottle accessibility status. A Pearson Chi-Squared test confirmed a highly significant relationship between bottle accessibility and stream height (seen in Table 3) of the drinking water fountains in Berkeley. This provides evidence that by restoring water fountain stream heights, many will become water bottle accessible. Some exceptions may apply depending on basin shape and depth, which wasn’t recorded in this study.

Only about 58% of the water fountains were reported as handicap accessible. Additionally, there was significant clustering (evident in Fig. 5) of non-handicap accessible fountains in the northern region of Berkeley and handicap accessible fountains in the western region. Surveyors were not trained in American Disabilities Act accessibility standards; therefore, the assessment was somewhat subjective. The relationship of handicap accessibility and terrain slope was not quantitatively examined in this study, however this was assessed by overlaying the fountain data on topographic maps (data not shown), and it was apparent that the cluster of in-accessible fountains were in the hills of north Berkeley. The significant regression association was likely a result of elevation slope as a confounding factor. The northern region of Berkeley has a relatively greater slope (Fig. 5) and a higher median income than the western and southern regions of Berkeley.

## Conclusions

There was no evidence that condition of fountains varied based on the race or socio-economic characteristics of households surrounding water fountains in the City of Berkeley. The results of this study can be used by the City to prioritize maintenance of the fountains as well as planning the locations of future fountain installations. The finding that fountains were primarily located in parks rather than streets with high pedestrian traffic, could be considered by City Planners as they endeavor to make the City more “walkable”, and attractive to residents and tourists.

Because Berkeley obtained notoriety as the first US city to institute a sweetened beverage tax, an opportunity existed to promote healthy alternatives such as tap water. This study showed that mobile-GIS technology is useful for generating information-rich maps of an important public resource. Having an easily accessible map that shows the locations and conditions of drinking water fountains can support the goal of promoting water consumption.

## Abbreviations

ACS: American Community Survey
GIS: Geographic information system
SES: Socio-economic status
